# Supplementary material for: Dynamics of COVID-19 under social distancing measures are driven by transmission network structure
Source: medRxiv. 2021 Jan 15:2020.06.04.20121673. Originally published 2020 Jun 5. Preprint. [Version 2] doi: 10.1101/2020.06.04.20121673 (PMC7302300; doi:10.1101/2020.06.04.20121673)
Supplement: 1 [file NIHPP2020.06.04.20121673-supplement-1.pdf]

medRxiv preprint doi: https://doi.org/10.1101/2020.06.04.20121673; this version posted January 15, 2021. The copyright holder for this preprint (which was not certified by peer review) is the author/funder, who has granted medRxiv a license to display the preprint in perpetuity.
It is made available under a CC-BY 4.0 International license .

Supplementary Methods

Model of COVID-19 spread and clinical progression

We use a compartmental epidemiological model, based on the classic SEIR model, to describe the spread and clinical progression of COVID-19 (**Figure 2**). The basic model structure is inspired by many studies on the natural clinical progression of COVID-19 infection. Infected individuals do not immediately develop severe symptoms, but instead pass through milder phases of infection first. For a nice summary, see Wu and McGoogan [88]. Susceptible (S) individuals who become infected start out in an exposed class E, where they are asymptomatic and do not transmit infection. Progression from the exposed stage to the infected stage I, where the individual is symptomatic and infectious, occurs after an average duration of $T_E$days. The clinical descriptions of the different stages of infection are given below. Infected individuals begin with *mild* infection (I_1) that lasts on average T_1 days, from which a fraction (f_1)progress to *severe* infection (I_2) as opposed to recovering. Severe infection progresses to a *critical* stage (I_3) in a portion (ef_2) of individuals and resolves in the rest, after an average duration T_2. A fraction (f_3) of individuals with critical infection die, while the remainder recover. The average duration of critical infection is T_3 days. Recovered individuals are tracked by class R and are assumed to be protected from re-infection for the duration of the epidemic. Individuals may transmit the infection at any stage, though with different rates. The transmission rate in stage *i*, β_i.

The clinical definitions of the infection stages are as follows

- Mild infection - These individuals have symptoms like fever and cough and may have mild pneumonia. Hospitalization is not required (though in many countries such individuals are also hospitalized)
- Severe infection - These individuals have more severe pneumonia that leads to dyspnea, respiratory frequency <30/min, blood oxygen saturation <93%, partial pressure of arterial oxygen to fraction of inspired oxygen ratio <300, and/or lung infiltrates >50% within 24 to 48 hours. Hospitalization and supplemental oxygen are generally required.
- Critical infection - These individuals experience respiratory failure, septic shock, and/or multiple organ dysfunction or failure. Treatment in an ICU, often with mechanical ventilation, is required.

A summary of the variable definitions:
- S: Susceptible individuals
- E: Exposed individuals - infected but not yet infectious or symptomatic
- I_i: Infected individuals in severity class *i*. Severity increases with *i* and we assume individuals must pass through all previous classes
    - I_1: Mild infection
    - I_2: Severe infection
    - I_3: Critical infection
- R: Individuals who have recovered from disease and are now immune
- D: Dead individuals

medRxiv preprint doi: https://doi.org/10.1101/2020.06.04.20121673; this version posted January 15, 2021. The copyright holder for this preprint (which was not certified by peer review) is the author/funder, who has granted medRxiv a license to display the preprint in perpetuity.
It is made available under a CC-BY 4.0 International license .

- $N=S+E+I_1+I_2+I_3+R+D$ Total population size (constant)

To describe the average time course in a large, well-mixed population, the model can be represented by the following set of differential equations:

$$
\begin{aligned}
\dot{S} &= -(\beta_1 I_1 + \beta_2 I_2 + \beta_3 I_3) S \\
\dot{E} &= (\beta_1 I_1 + \beta_2 I_2 + \beta_3 I_3) S - E/T_E \\
\dot{I}_1 &= E/T_E - I_1/T_1 \\
\dot{I}_2 &= f_1 I_1/T_1 - I_2/T_2 \\
\dot{I}_3 &= f_2 I_2 - I_3/T_3 \\
\dot{R} &= (1-f_1) I_1/T_1 + (1-f_2) I_2/T_2 + (1-f_3) I_3/T_3 \\
\dot{D} &= f_3 I_3/T_3
\end{aligned}
$$

The basic reproductive ratio R_0 of this model is:

$$R_0 = N\beta_1 T_1 + N f_1 \beta_2 T_2 + N f_2 \beta_3 T_3$$

Our model makes several assumptions with regards to the clinical and epidemiological dynamics of COVID-19. We do not explicitly track asymptomatic infections, which have been estimated to be around 20% of infections [34] (but estimates range from 1% to 50%, reviewed in [89]). However, in our model asymptomatic infections can be considered to be part of the “mild infections” compartment. There is significant evidence that asymptomatic individuals are infectious, and with this assumption their infectiousness would be equal to those with mild infection. For simplicity we assume that critical infection always occurs after passing through a stage of severe infection, while in reality there appears to be some individuals who progress directly from mild to critical infection. Similarly, we assume that only individuals who have progressed to critical infection die, though there is now some evidence that death can occur unexpectedly in individuals who are not already hospitalized. We do not think that any of these assumptions affect the main conclusions of our paper, which does not focus on hospital resource use or morbidity/mortality estimates. In general our model allows for individuals at all stages of infection to transmit to others. However, for this paper we have assumed that only individuals with mild infection can transmit. We think it’s likely that an individual is most infectious during this stage, when they would still be in the community and feeling well enough to interact with others. We thus ignore transmission from hospitalized patients to their healthcare providers ($\beta_2=\beta_3=0$). We make the standard SEIR model assumption that the time until an infected individual experiences symptoms is the same as the time until they become infectious, whereas it has now been shown for COVID-19 that there is significant transmission risk starting ~1 day before symptom onset. While this disease feature is important for studies that examine symptom based case detection/isolation, for simplicity we have ignored this feature in our model, which doesn’t focus on these issues. The serial interval produced by our model parameters agrees with observed values, and so we believe our timescales for infections spread are realistic. We do not explicitly consider age-dependent rates of infection or progression to more serious stages.

medRxiv preprint doi: https://doi.org/10.1101/2020.06.04.20121673; this version posted January 15, 2021. The copyright holder for this preprint (which was not certified by peer review) is the author/funder, who has granted medRxiv a license to display the preprint in perpetuity.
It is made available under a CC-BY 4.0 International license .

The differential equation version of our model is available as an online simulation tool in which users can explore the effects of these assumptions of model structure as well as parameter values on model outputs: https://alhill.shinyapps.io/COVID19seir/

Stochastic network implementation

To avoid the assumptions inherent in formulating an epidemiological model using standard differential equations (e.g. assuming a randomly-mixing population, deterministic dynamics, and exponentially-distributed durations of each infection stage), we implement the model as a stochastic process simulated on a transmission network.

We believe that this implementation is important for our research questions, for a few main reasons. Firstly, respiratory infections are spread by close physical contact that occurs in highly structured contact networks, which impact the growth rate of outbreaks, the relationship between an individual's inherent infectiousness and the basic reproductive ratio R_0, the variability in secondary infections between individuals, and the effects of control measures on spread. Secondly, typical differential equation models implicitly assume that the durations of infection stages are extremely long-tailed, which can lead to unrealistic conclusions about the timescale of the epidemic response to control measures as well as to the relationship between the early growth rate, serial interval or generation time, and R_0. Finally, we believe it is important to model the uncertainty in epidemic trajectories that arises from the inherent stochasticity of transmission.

In our stochastic formulation, we assumed that the duration of each stage of infection was gamma-distributed, with both the mean and variance taken from the literature. Individuals were connected with a fixed, weighted contact network that determined the potential paths of transmission. The network is represented as a sparse matrix to save memory (a list of the index of each node in an edge, along with the edge weight). The model was implemented with a discrete time stochastic process that tracked the state of each individual and the time since they first entered that state. The model was implemented in Python using JAX, a framework for generating high-performance code optimized to run on GPUs. Our code was entirely run in the cloud on Google Colab. The code is available at :
https://github.com/alsnhll/COVID19NetworkSimulations

Model parameters

We estimated the distribution of the duration of each stage of infection from the literature. For the incubation period, we used an estimate of 5 ± 4 days from [34,90], which is consistent with most other estimates. The duration of mild infection, which we assume is roughly equal to the infectious period, can be estimated from a few different sources: a) the duration of mild symptoms, b) the time from symptom onset to hospitalization (e.g. time to progress to severe stage), or c) the duration of viral shedding via sputum or throat swabs, d) inferred from both the incubation period and the "serial interval" between symptom onset in an index case and a secondary case they

medRxiv preprint doi: https://doi.org/10.1101/2020.06.04.20121673; this version posted January 15, 2021. The copyright holder for this preprint (which was not certified by peer review) is the author/funder, who has granted medRxiv a license to display the preprint in perpetuity.
It is made available under a CC-BY 4.0 International license .

infect. Considering (d), we took an estimate of the serial interval from Bi et al [34], who used a large cohort of transmission pairs and reported values separately for situations where the index case was not rapidly isolated or hospitalized during mild infection. Similar values were found in [69–72]. Some other studies have estimated smaller values (e.g. [51]), likely due to rapid isolation of symptomatic cases in Asia [70]. This value implies an infectious period of 6 ± 2 days. This is relatively consistent with estimates of the time from symptom onset to hospitalization [52,91–93], and is roughly equivalent to the time at which average viral loads drop 4-5 orders of magnitude from peak values [94].

The duration of severe infection in our model is equivalent to the time from hospital admission to recovery for individuals who did not progress to the critical stage, or the time from hospital admission to ICU admission (since critical cases require ICU-level care). Since it is hard to find direct estimates of this duration, we instead used estimates of the total time from symptom onset to ICU-admission (e.g. combined length of mild + severe infection) [92,93], and subtracted the inferred duration of mild infection. This led to an estimate of 6 ± 4.5 days. We estimated the duration of critical infection (the length of ICU stay) directly from cohort studies to be 8 +/- 6 days [92,95]. With these estimates, we verified that the total time from symptom onset to death/recovery agreed with data (~20 ± 10 days) [69,92,96]. Note that we assumed that the duration of each stage of infection was independent of the previous stage and was also independent of the eventual outcome.

In each stage of infection, an individual can either progress to the next stage or recover, and the probability of each was also estimated from the literature [8,88,97]. These large clinical cohort studies estimated that ~ 20% of infections required hospitalization (i.e. progress to severe stage), ~5% require ICU care (i.e. progress to critical stage), and an overall case fatality risk of 2%. This leads to probabilities of progression of 0.2 from $I_1 \rightarrow I_2$, 0.25 from $I_2 \rightarrow I_3$, and 0.4 from $I_3 \rightarrow D$. However, there is significant uncertainty in these values. More recent estimates suggest the rates of progression to more serious stages of infection might be lower after correcting for asymptomatic/undiagnosed cases in China [96], but in contrast, recent studies from the US suggest higher rates of ICU admission and death among hospitalized patients compared to in Asia [98,99]. Since the results of our paper focus on the timing of the peaks of different infection stages but not on the prevalence at these peaks, they are not sensitive to these assumptions.

We chose a value of the transmission rate β such that the early epidemic doubling time was ~ 4 days (growth rate r = 0.17/day) and the basic reproductive rate R_0 = 3. While some studies have estimated R_0 values as low as 2, these have generally used estimates based only on the serial interval which are mathematically problematic, used short estimates of the serial interval that are influenced by rapid case isolation in Asia as opposed to longer values found in other studies that correct for this, and fit to longer doubling times that what was observed early in the outbreak in the US and Europe. For a simple unweighted network, we estimate β using the formula $R_0 = \beta T_1 (n - 1)$ where, n is the mean degree of the network and T_1 is the average duration of mild infections. This relationship is modified for the weighted two-layer network,

medRxiv preprint doi: https://doi.org/10.1101/2020.06.04.20121673; this version posted January 15, 2021. The copyright holder for this preprint (which was not certified by peer review) is the author/funder, who has granted medRxiv a license to display the preprint in perpetuity.
It is made available under a CC-BY 4.0 International license .

$$R_0 = \beta T_1(w_{HH}n_{HH} + w_{EX}n_{EX} - (w_{HH}f_{HH} + w_{EX}f_{EX}))$$

where, $f_{HH} = \frac{w_{HH}n_{HH}}{w_{HH}n_{HH}+w_{EX}n_{EX}}$ and $f_{EX} = 1 - f_{HH}$ with the weight and mean degree of the household (external) layer given by w_{HH} (w_{EX}) and n_{HH} (n_{EX}). As this equation suggests, the rate of infection is constant per time per contact for a given contact type, and hence would be described as "density-dependent" transmission.

The time at which we implemented interventions in our simulations was chosen to occur at a realistic time in the epidemic relative to real responses to COVID-19. To estimate this time for a typical US metro area, we downloaded case and death counts for each US county and aggregated them into the top 50 largest metro areas. Then we collected the time that stay-at-home orders were implemented in each of these regions, and looked at the median cumulative case and death counts by that day, and chose our intervention time to match those values. We found that there were on average around 200 cases per million inhabitants at time of stay at home intervention (range [30,1000]), and ~5 [0-20] deaths per million. We assumed that reported "cases" are hospitalized individuals only (cumulative I_2). These values were recreated in our simulation in well-mixed populations on Day 40, which we used as the intervention time. At this time, ~0.5% of the population had ever been infected (including E, I_1, I_2, I_3, R, and D). Since the median day shelter-in-place orders were implemented across these metros was April 1, Day 0 in our simulation corresponds to ~ Feb 20. For simulations in other networks, we kept R_0 constant but due to the population structure, the exponential growth rate of the epidemic varied between different values of the household and external contact structure. Thus for each structure we chose slightly different intervention start times to keep the infection prevalence consistent across comparisons. The start times varied from Day 43 (when w_{EX}/w_{HH} = 3, 1), Day 45 (when w_{EX}/w_{HH} = 1/3) to Day 55 (when w_{EX}/w_{HH} = 1/9).

medRxiv preprint doi: https://doi.org/10.1101/2020.06.04.20121673; this version posted January 15, 2021. The copyright holder for this preprint (which was not certified by peer review) is the author/funder, who has granted medRxiv a license to display the preprint in perpetuity.
It is made available under a CC-BY 4.0 International license .

Table 1: Model parameters for duration of each stage of infection

Infection stage		Symbol	Duration (days) Mean	STD	Source
Exposed (incubation period)	E	T_E	5	4	[34,90]
Mild infection	I_1	T_1	6	2	Estimated to agree with serial interval and [52,91–94,100]
Severe infection	I_2	T_2	6	4.5	Estimated to agree with time to ICU admission
Critical infection	I_3	T_3	8	6	Estimated to agree with [92,95] and time to recovery/death
Serial interval			8	4.5	Derived from above, agrees with [34,69–72]
Hospitalization (avg, w or w/o ICU stay)			8	6	Derived from above
Hospital (incl. ICU stay)			14	7.5	Derived from above
Symptom onset to ICU admission			12	5	[92,93]
Symptom onset to death or recovery			20	10	[69,92,96]

medRxiv preprint doi: https://doi.org/10.1101/2020.06.04.20121673; this version posted January 15, 2021. The copyright holder for this preprint (which was not certified by peer review) is the author/funder, who has granted medRxiv a license to display the preprint in perpetuity.
It is made available under a CC-BY 4.0 International license .

Table 2: Model parameters for probability of progressing from each stage of infection

Infection stage		Probability of progression		
		**Symbol**	**Value**	**Source**
Exposed (incubation period)	E		1	
Mild infection	I_1	f_1	0.2	[8,88,97]
Severe infection	I_2	f_2	0.25	[8,88,97]
Critical infection	I_3	f_3	0.4	[8,88,97]

Well-mixed network structure

We approximated a well-mixed population by randomly connecting each individual to 100 other individuals in the population. While a truly “well-mixed” network would connect every one to everyone else, with the large population sizes we use (10^6), this would require huge amounts of memory and negate the computational efficiency of using sparse matrices to represent the networks. Since in reality each individual will only transmit to a few others before recovering, any uniform random network with a large degree is a good approximation to a fully-connected network.

Two-layer household network structure

Construction: We constructed a weighted two-layer network, consisting of a layer for within-household connections and another for external connections. Individuals were first assigned households using the distribution of household sizes in the United States (data obtained from the 2010 census, mean household size $n_{HH} \sim 2.5$). All individuals in a household were connected to each other. Each individual was then assigned random external contacts. The degree distribution for external connections was obtained from contact survey data that recorded daily interactions of individuals [20,21]. This data was originally collected for a subset of countries and then projected to other countries using a wide range of demographic data. The contacts are age structured and include the type, duration, location and frequency of the contact. These surveys were designed to capture interactions relevant to the spread of respiratory diseases, and include interactions consisting of either physical contact or close face-to-face conversations. From this data, we obtained daily non-household contacts by summing ‘work’, ‘school’ and ‘other’ contacts. For the United States there were on average $n_{EX} \sim 7.5 \pm 2$ (mean $\pm$ standard deviation) daily non-household contacts, equivalent to 3x household contacts. The variance was obtained by the variance in the mean across all age groups. We assigned external degrees to individuals following a binomial distribution with this

medRxiv preprint doi: https://doi.org/10.1101/2020.06.04.20121673; this version posted January 15, 2021. The copyright holder for this preprint (which was not certified by peer review) is the author/funder, who has granted medRxiv a license to display the preprint in perpetuity.
It is made available under a CC-BY 4.0 International license .

mean and standard deviation. This assignment was done randomly and did not depend upon the size of the person's household. The network layer was created by giving 'stubs' to individuals equal to their external degrees. These stubs were then randomly paired between individuals in the population to create connections. In addition to the number of contacts, the probability of infection also depends upon the duration and intensity of the contact. We assigned weights (w_{HH}, w_{EX}) to the two layers of the network to account for this. We considered different scenarios (keeping R_0 fixed) where, $w_{HH} = 1$ and varied $w_{EX} = 3, 1, 1/3, 1/9$ to reflect the relative importance between household and external contacts.

In order to investigate the robustness of our results with respect to details in the large-scale clustering of the network, we also constructed a hierarchically structured external layer using ideas from nested metapopulation models [101,102]. Individuals were assigned to 'neighborhoods' of size 10,000 (there are 100 such neighborhoods for the population size of 1 million) and as an extreme case 90% of their external connections were forced to be within the same neighborhood with $n_{EX} \sim 7.5 \pm 2$ as before.

<u>Intervention:</u> Intervention, corresponding to social distancing measures, was modeled by reducing the weight of the external layer, $w'_{EX} = (1 - \varepsilon)\, w_{EX}$ where, $\varepsilon\ (0 \le \varepsilon \le 1)$ is the 'intervention efficacy'. When we are in the parameter regime where the probability an individual infects any given external contact over the duration of their infectious period is significantly less than one (i.e. $R_0^{EX} < n_{EX}$), reducing the weight of external contacts is equivalent to randomly removing external contacts (i.e. $n'_{EX} = (1 - \varepsilon)\, n_{EX}$). Only in the regime where the transmission rate per contact is so high that R_0^{EX} is limited only by the number of contacts would these two alternative methods of implementing social distancing lead to different results. Household contacts were either unaffected ($w'_{HH} = w_{HH}$) or doubled ($w'_{HH} = 2\, w_{HH}$) during intervention, to represent the increased time spent with household members.

<u>Partial relaxation of intervention</u>: We modeled a scenario of partial relaxation of social distancing measures in which every household could "merge" with another household. This relaxation altered the household layer so that all households in the network were paired with another random household and the two were merged to create a fully-connected joint household ($n'_{HH} \sim 5$). We considered two scenarios for how the external layer changed during partial relaxation of intervention. In the baseline scenario, the external layer was the same as during intervention. In a second scenario, the number of external contacts per individual was reduced so that the average number of total contacts was the same before and after merging ($n'_{EX} = n_{EX} - n_{HH}$). This partial relaxation was only conducted for the scenario where household and external connections had the same weight ($w_{HH} = 1$ and $w_{EX} = 1$).

<u>Individual probability of infection as a function of household size:</u> We computed the individual probability of infection retrospectively, after the epidemic had died out, for the case of a 100% effective intervention. We counted the number of individuals who were ever infected by the end of the epidemic and calculated the corresponding distribution of their household sizes. This is a

medRxiv preprint doi: https://doi.org/10.1101/2020.06.04.20121673; this version posted January 15, 2021. The copyright holder for this preprint (which was not certified by peer review) is the author/funder, who has granted medRxiv a license to display the preprint in perpetuity.
It is made available under a CC-BY 4.0 International license .

conditional probability that gives the probability of household size, given that an individual was infected P(HH size|inf). The probability of infection given household size was then calculated using Bayes' rule, P(inf|HH size) = P(HH size|inf) * P(inf)/P(HH size) where, P(inf) is the fraction of the total population that was infected during the epidemic and P(HH size) is the distribution of the household sizes in the population. The probability was averaged over 10 iterations of the simulation.

Five-layer network structure

We constructed a non-weighted 5-layer (1 household and 4 external) network for an age-structured population with realistically structured external layers. The population was divided into four age groups: preschool aged (ages 0-5), school-aged (ages 5-19), working-aged (ages 19-64) and elderly (ages 65+). The distribution of ages in the United States was obtained from the 2018 World Bank Age Structure data which consists of the population divided into 5-year age groups. In addition to the broad age groups, we also kept track of the 5-year age group that an individual belonged to. The contact survey data [21] used to construct the 2-layer network was also predominantly used here to determine the statistical properties of the layers.

Household layer: The household layer was constructed in the same manner as in the 2-layer network. Individuals were assigned to fully-connected households using the distribution of household sizes in the United States from the 2010 census data (mean household size, $n_{HH} \sim 2.5$).

School layer : The school layer contained connections between the school-aged age group in the population. This external layer was constructed using the technique described by Ball et al [103], where a network layer with a given degree distribution and correlations among connections (i.e. "clustering" or "transitivity") can be constructed. The degree distribution in this layer was obtained from the school contact survey data [21], which estimated that the school-going population in the United States, on average, had $n_{SCHOOL} \sim 7.3 \pm 1.8$ school-related contacts daily. The variance was obtained by the variance in the mean across the school-aged (5-19) age groups. Individuals were assigned degrees randomly from a binomial distribution with this mean and standard deviation. The age-structure of contacts at school suggests a significant level of clustering by age group. To create correlated connections, the method of Ball et al requires two additional parameters: The number of groups n to divide the population into, and r, which is roughly the extent to which individuals within the same group are connected. $r = 1$ corresponds to individuals being connected only within their group whereas $r = 0$ implies completely random connections without any correlation with members of their own group. Averaged over the school age groups (5-19), we found that $\sim 57\%$ of school contacts for an individual belonged to their own age group. Since there are three 5-year age groups in our school-aged population, we divided our population into those three groups ($n = 3$) and chose $r = 0.57$.

medRxiv preprint doi: https://doi.org/10.1101/2020.06.04.20121673; this version posted January 15, 2021. The copyright holder for this preprint
(which was not certified by peer review) is the author/funder, who has granted medRxiv a license to display the preprint in perpetuity.
It is made available under a CC-BY 4.0 International license .

Work layer : The work layer consisted of connections between the working-aged group in the population. This layer was constructed similarly to the school layer. We obtained the mean and standard deviation in number of daily work contacts from the contact survey data (5.0 ± 2.3). The variance was obtained by the variance in the mean across working-aged (20-64) age groups. Individuals were randomly assigned a work-place degree from a binomial distribution with the mean and standard deviation. Then we used a separate study by Potter et al that mapped real work-place networks to estimate the level of clustering (transitivity), at ~0.1 [15]. Note that Potter et estimated a higher degree, since their network contained *total* contacts in a work network and not just the daily contacts as given by the contact survey data, but the coefficient of variation is similar. We again used the Ball et al technique [103] to create clustering in the network. High transitivity can be achieved by choosing appropriate correlation parameters n and r. We assumed this clustering arose from the fact that people belonging to the same work-place have a higher chance of being connected to each other. According to NAICS (North American Industry Classification System), $\sim 80\%$ of businesses in the United States have ≤ 10 employees and so for the sake of simplicity, we assigned each individual to a 'work-place' of size ten ($n = \frac{\#\ of\ working\ individuals}{10}$). We picked $r = 0.8$ in order to have a high level of transitivity (~ 0.12) while also ensuring inter work-place connections.

Friend (social contacts) layer: The friend layer represents social contacts of people, their 'friend circles'. Individuals were assigned to fully-connected friend groups, where friend groups consisted of people belonging to the same age group (preschool, school, working, elderly). The distribution of sizes of these friend groups were obtained from Wrzus et al [27], which is a meta-analysis of the effects of age on social networks. From their data, we estimated that the mean sizes of friend groups for individuals less than 20 years of age is ~ 10, that for the working age population ~ 7 and ~ 5 for the elderly. These constitute *total* social contacts for the individuals and not just their daily interactions as given by the contact survey data. The 'other' category in the contact survey data [21] gives the number of daily interactions that an individual has that are not within the household, at school or at the work-place. In our network, the friend layer and the community layer together constitute this 'other' category. According to the data, averaged over the age groups, there are $\sim 4.3 \pm 1.9$ such interactions for an individual in the United States. The variance was obtained by the variance in the mean across all age groups. Since the total social contacts are much higher than this daily value, we scaled the sizes of the friend groups ($n_{FRIEND} \rightarrow n_{FRIEND}/4$) to reflect daily social contacts. The scaling factor was chosen to ensure that the community layer didn't end up being too sparse. We chose to do this instead of creating a weighted network for the sake of simplicity. The friend group sizes were drawn using the negative binomial distribution to account for the large variance (estimated variance post scaling: 6.25, 2.5, 1.75 for the three age-groups) seen in the data.

Community layer: This layer constitutes the additional random contacts an individual has during the course of their day. As mentioned previously, together with the social layer this constitutes the 'other' category in the contact survey data. We chose the average degree for this layer, $n_{COMM} \sim 1.72 \pm 0.76$ such that the combined degree for the two layers matches what is seen in

medRxiv preprint doi: https://doi.org/10.1101/2020.06.04.20121673; this version posted January 15, 2021. The copyright holder for this preprint (which was not certified by peer review) is the author/funder, who has granted medRxiv a license to display the preprint in perpetuity.
It is made available under a CC-BY 4.0 International license .

the data. We used a binomial distribution and assigned degrees randomly to the individuals. This layer was constructed in the same manner as the external layer in the 2-layer network. It amounts to using the Ball et al [103] technique with $n = 1$ and $r = 0$.

Sensitivity analysis: We introduced the idea of "neighborhood" clustering to check the robustness of the results to clustering of contacts at an intermediate-scale between households and the city as a whole. As in the 2 layer network, individuals were assigned to neighborhoods of size 10,000. School and community layer contacts for individuals were predominantly within their neighborhood. The community layer was created by enforcing 90% ($r = 0.9$) of the connections to be within the same neighborhood ($n = 100$) with $n_{COMM} \sim 1.72 \pm 0.76$. School-aged individuals from each neighborhood, belonging to the same 5 year age group, were assigned to schools of size 500 (~ average size of public schools in the US according to the 2017 data from the National Center for Education Statistics). The degree distribution of the school contacts was the same as before, $n_{SCHOOL} \sim 7.3 \pm 1.8$ but all school contacts for an individual were restricted to their own school ($n = \frac{\#\, school\text{-}aged}{500}$, $r = 1$).

Intervention : The social distancing intervention was modeled by deleting a certain percentage of external connections. School connections were always completely deleted during intervention, whereas social and community connections were deleted depending upon what we termed as the 'efficacy' of intervention. For example, 85% efficacy corresponds to deleting 85% of contacts in these layers. We considered two scenarios for the effect of intervention on the work-layer. In one case, similar to the social and community layer, a percentage of work contacts was deleted depending upon the intervention efficacy. Random deletion of connections leads to a large reduction in the transitivity of the work layer, from ~ 0.12 to ~ 0.02. In the second case, the effect of intervention was modeled as a clustered deletion of workplaces instead. For example, 85% intervention efficacy corresponds to 85% of workplaces being dissolved. Clustered deletion still maintains a high level of transitivity ~ 0.10 in the work layer. Note as all school connections were completely deleted during intervention, the total effective efficacy of intervention was a bit higher than that for the social, community and work layers. For example, 85% efficacy in these external layers corresponds to a total efficacy of 88%.

Probability of infection for an individual working during intervention: We calculated the probability of infection for two intervention efficacies (85%, 70%) at day 300 after the start of the outbreak. We first obtained the fraction of the infected people (at day 300) who were still working during the intervention. This gives the conditional probability that an individual was working during intervention given that they were infected, P(working|inf). We calculated the probability of infection given that an individual was working during intervention using Bayes' rule, P(inf|working) = P(working|inf) * P(inf)/P(working) where, P(inf) is the fraction of the total population that was infected by day 300 of the epidemic and P(working) is the fraction of the total population that was working during intervention. The probability was averaged over 10 iterations of the simulation.

medRxiv preprint doi: https://doi.org/10.1101/2020.06.04.20121673; this version posted January 15, 2021. The copyright holder for this preprint (which was not certified by peer review) is the author/funder, who has granted medRxiv a license to display the preprint in perpetuity.
It is made available under a CC-BY 4.0 International license .

Probability of infection for an individual living with working household members during intervention: This was also calculated at day 300 of the epidemic for the two intervention efficacies and, averaged over 10 iterations of the simulation. We obtained the fraction of the infected people (at day 300) who had at least 1 household member who was working during intervention. This corresponds to the probability that an infected individual had at least one working household member during intervention, P(HH working|inf). As before, the probability of infection given that an individual was living with working household members during intervention was then calculated using Bayes' rule, P(inf| HH working) = P(HH working|inf) * P(inf)/P(HH working) where, P(inf) is the fraction of the total population that was infected by day 300 of the epidemic and P(HH working) is the fraction of the total population that had at least one working household member during the intervention.

Probability of infection for a non-working individual with no working household members during intervention: This was calculated in the same way as the other probabilities. We obtained the fraction of the infected people (at day 300) who were not working and had no working household member, P(HH not working|inf). As before, the required probability was then calculated using Bayes' rule, P(inf| HH not working) = P(HH not working|inf) * P(inf)/P(HH not working) where, P(inf) is the fraction of the total population that was infected by day 300 of the epidemic and P(HH not working) is the fraction of the total population that was not working and had no working household members during the epidemic.

COVID-19 Data

Wuhan, China: Data from Wuhan, China was obtained from the two daily situation reports published each day: http://wjw.wuhan.gov.cn/ztzl_28/fk/yqtb/index_11.shtml. We extracted new cases, new deaths, the number of individuals currently hospitalized with severe infection (which we took as a proxy for "hospitalized") and the number of individuals currently hospitalized with "critical" infection (which we took as a proxy for individuals in the ICU). Data was already digitized by Li et al [104] for January and February (https://github.com/c2-d2/COVID-19-wuhan-guangzhou-data); we added data into March. For some dates the numbers of severe and critical cases were only reported for the entire province of Hubei, and in those instances, following Li et al, we assumed the proportion of those attributable to the city of Wuhan was equal to the proportion of currently active cases occurring in Wuhan. The vast majority of the Hubei outbreak took place in Wuhan.

Lombardy, Italy: Data on daily new cases and deaths as well as current patients hospitalized or in ICU for the Lombardy region of Italy was downloaded from Github repository maintained by the Department of Civil Protection in Italy : https://github.com/pcm-dpc/COVID-19
`

Madrid, Spain: Data for daily new cases, daily deaths, daily new hospital admissions, and daily new ICU admissions were obtained from an online application maintained by the National Center for Epidemiology, using data from the Ministry of Health:

medRxiv preprint doi: https://doi.org/10.1101/2020.06.04.20121673; this version posted January 15, 2021. The copyright holder for this preprint (which was not certified by peer review) is the author/funder, who has granted medRxiv a license to display the preprint in perpetuity.
It is made available under a CC-BY 4.0 International license .

https://cnecovid.isciii.es/covid19/. In the “documentation and data” section of the application there is a link to a .csv file with all the data used in the web app.

New York City, New York, USA: Daily new cases and deaths were downloaded from the Github repository of the New York City Department of Health and Mental Hygiene: https://github.com/nychealth/coronavirus-data. Number of currently individuals hospitalized or in ICU by date was obtained from the Github repository for digital news platform The City, who obtains this data directly from the New York state governor’s office: https://github.com/thecityny/covid-19-nyc-data

Los Angeles County, California, USA: Daily new cases and deaths were obtained from the Github repository maintained by the Los Angeles Times newspaper: https://github.com/datadesk/california-coronavirus-data. The number of individuals currently in the hospital or ICU was obtained from the California Health & Human Services Open Data Portal: https://data.chhs.ca.gov/dataset/california-covid-19-hospital-data-and-case-statistics/resource/6cd8d424-dfaa-4bdd-9410-a3d656e1176e?view_id=b23b0158-a85d-4bf2-95b1-96f7556f7342

medRxiv preprint doi: https://doi.org/10.1101/2020.06.04.20121673; this version posted January 15, 2021. The copyright holder for this preprint (which was not certified by peer review) is the author/funder, who has granted medRxiv a license to display the preprint in perpetuity.
It is made available under a CC-BY 4.0 International license .

Supplementary Figures

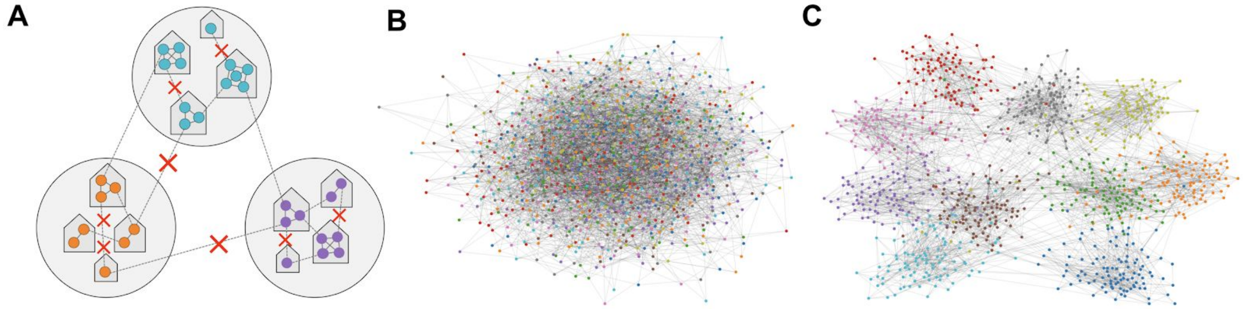

Figure S1: Creating hierarchical structuring in transmission networks via "neighborhoods". A) Schematic of the construction of the networks neighborhood clustering. Each household belongs to a mutually-exclusive "neighborhood", and external connections are preferentially created within the same neighborhood. B-C) Visualizations of connections in the external layer of the two-layer transmission network with and without neighborhood clustering**.** For ease of visualization, this is plotted for a population size of 1000 with 10 "neighborhoods" (node color) of size 100. In the real simulations, the population size is 10^6 with 100 neighborhoods of size 10,000. A) The external layer consists of random connections between individuals, irrespective of their neighborhoods. B) 90% of external connections are preferentially attached to individuals within their own neighborhood, creating more intermediate-scale clustering in the network.

medRxiv preprint doi: https://doi.org/10.1101/2020.06.04.20121673; this version posted January 15, 2021. The copyright holder for this preprint (which was not certified by peer review) is the author/funder, who has granted medRxiv a license to display the preprint in perpetuity.
It is made available under a CC-BY 4.0 International license .

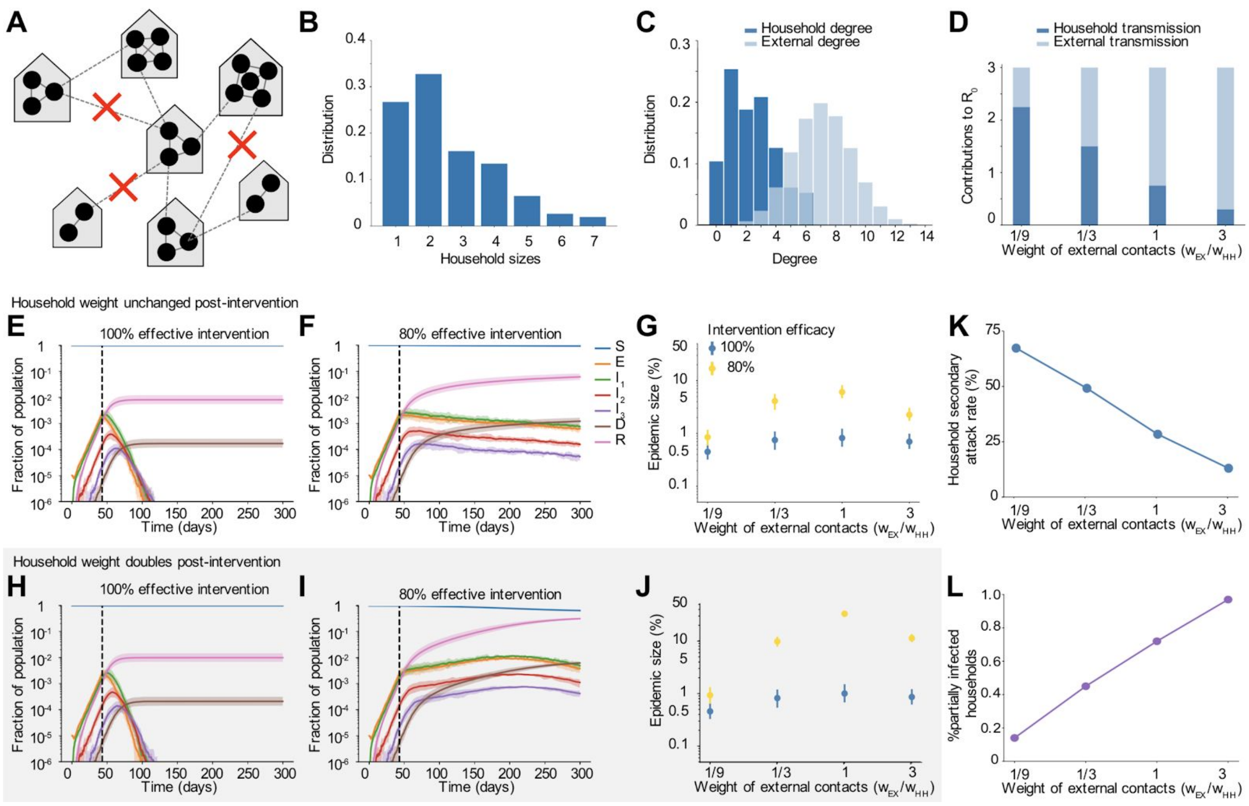

Figure S2. Dynamics pre and post social distancing interventions in network-structured populations with household and external transmission, with neighborhood clustering. A) Multi-layer network of transmission. Individuals have contacts within their households and with others outside the household, which preferentially occur within a local neighborhood (Figure S1). Household and external contacts may have different weights (e.g. different likelihood of transmission), due to for example different levels of physical contact or time spent together per day. Social distancing interventions (red X) remove or decrease the weight of external contacts. B) Distribution of household sizes. C) Distribution of the # of contacts (degree) within the household and outside the household. D) The contribution of household and external spread to the total R_0 value as a function of the relative weight of external contacts. E)-F) Simulated time course of different clinical stages of infection under an intervention with efficacy of 100% (E) or 80% (F) at reducing external contacts, when household and external contacts have equal weight. Black dotted line shows the time the intervention began. G) The role of the relative importance of household vs external contacts in determining the outcome of the intervention, measured by the size of the epidemic. Epidemic final size is defined as the percent of the population who have recovered by day 300. H-J) Same as above but under the scenario where the weight of household contacts doubles post-intervention ($w_{HH} \rightarrow 2w_{HH}$, due to increased time spent in house). K) The household secondary attack rate, defined as the probability of transmission per susceptible household member when there is a single infected individual in the house, as a function of the relative weight of external contacts. L) The percent of households which are "seeded" with infection at the time the intervention was implemented (i.e. have at least one infected individual). In all scenarios the overall infection prevalence at the time intervention was started was identical.

medRxiv preprint doi: https://doi.org/10.1101/2020.06.04.20121673; this version posted January 15, 2021. The copyright holder for this preprint (which was not certified by peer review) is the author/funder, who has granted medRxiv a license to display the preprint in perpetuity.
It is made available under a CC-BY 4.0 International license .

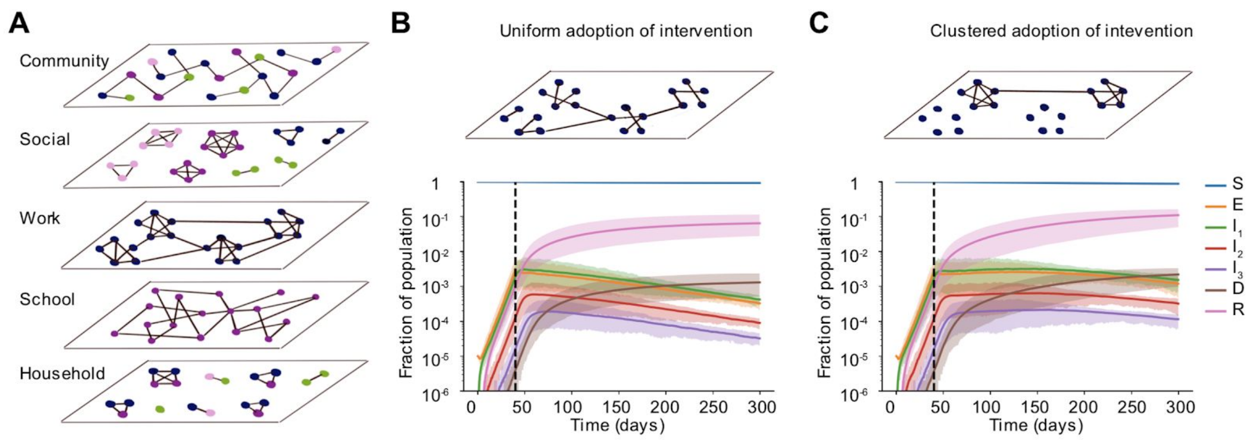

Figure S3: Clustered vs uniform adoption of social distancing measures, with lower efficacy. A) Schematic of the multi-layer network created to more realistically capture non-household contacts and how they are altered by social distancing measures. In each layer, the degree distribution and level of clustering were chosen to match data. The "community" layer represents any other contact not fitting in the other four categories. Colors of nodes represent four broad age groups that determine network membership and structure: preschool-aged (pink), school-aged (purple), working-aged (blue) and elderly (green). B) - C) Simulated time courses of infection in the presence of social distancing intervention with random (B) vs clustered (C) adherence to measures. In both cases, all school connections were deleted post-intervention and 70% of connections were uniformly deleted at random in the social and community layers. In B) 70% of work connections were uniformly deleted whereas, in C) 70% of workplaces were dissolved, leading to clusters of disconnected vs connected individuals in the work layer of the network. The effective intervention efficacy for all layers combined was ~ 75% in both scenarios. Black dotted line shows the time the intervention began.

medRxiv preprint doi: https://doi.org/10.1101/2020.06.04.20121673; this version posted January 15, 2021. The copyright holder for this preprint (which was not certified by peer review) is the author/funder, who has granted medRxiv a license to display the preprint in perpetuity.
It is made available under a CC-BY 4.0 International license .

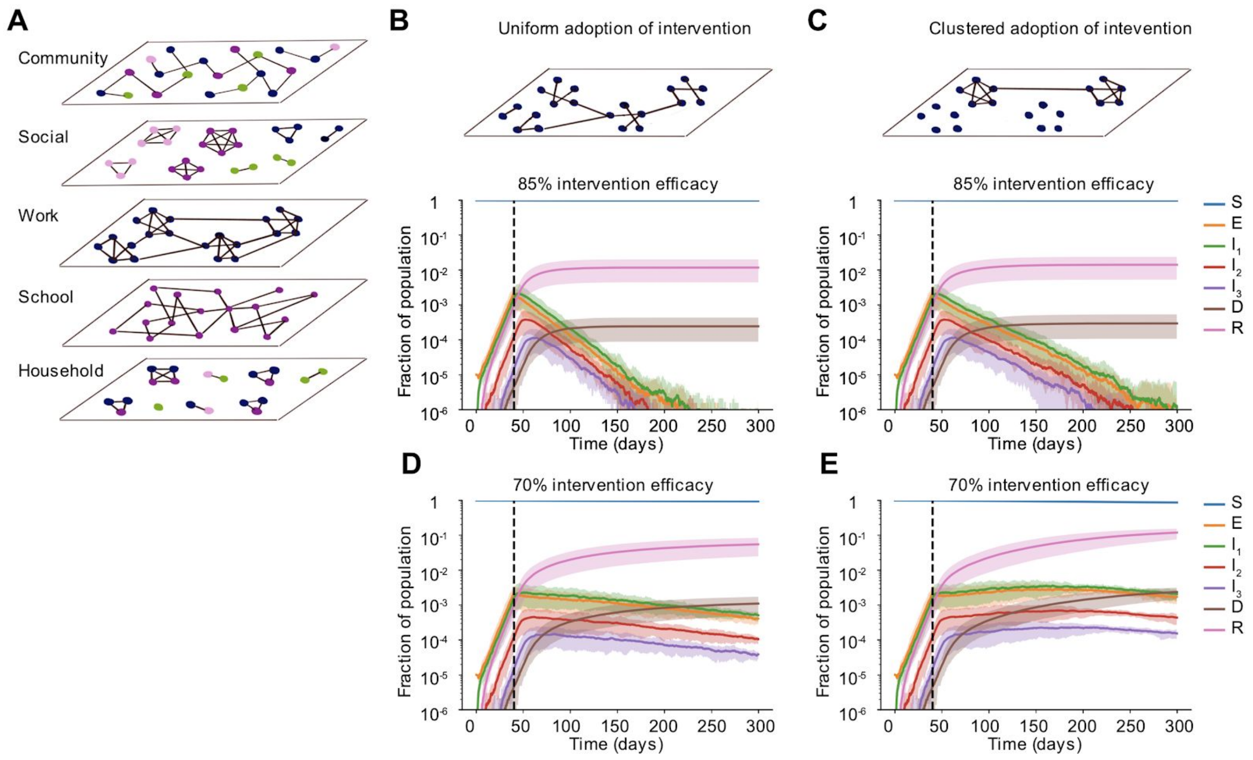

Figure S4: Clustered vs uniform adoption of social distancing measures, with neighborhood clustering. A) Schematic of the multi-layer network created to more realistically capture non-household contacts and how they are altered by social distancing measures. In each layer, the degree distribution and level of clustering were chosen to match data. The "community" layer represents any other contact not fitting in the other four categories. Colors of nodes represent four broad age groups that determine network membership and structure: preschool-aged (pink), school-aged (purple), working-aged (blue) and elderly (green). Community and school connections occurred within local neighborhoods. B) - E) Simulated time courses of infection in the presence of social distancing intervention with random (B,D) vs clustered (C,E) adherence to measures. In both cases, all school connections were deleted post-intervention and 85% (top) or 70% (bottom) of connections were uniformly deleted at random in the social and community layers. In B) 85% (top) or 70% (bottom) of work connections were uniformly deleted whereas, in C) 85% (top) or 70% (bottom) of workplaces were dissolved, leading to clusters of disconnected vs connected individuals in the work layer of the network. The effective intervention efficacy for all layers combined was ~ 88% (top) or ~75% (bottom) in both scenarios. Black dotted line shows the time the intervention began.

medRxiv preprint doi: https://doi.org/10.1101/2020.06.04.20121673; this version posted January 15, 2021. The copyright holder for this preprint (which was not certified by peer review) is the author/funder, who has granted medRxiv a license to display the preprint in perpetuity.
It is made available under a CC-BY 4.0 International license .

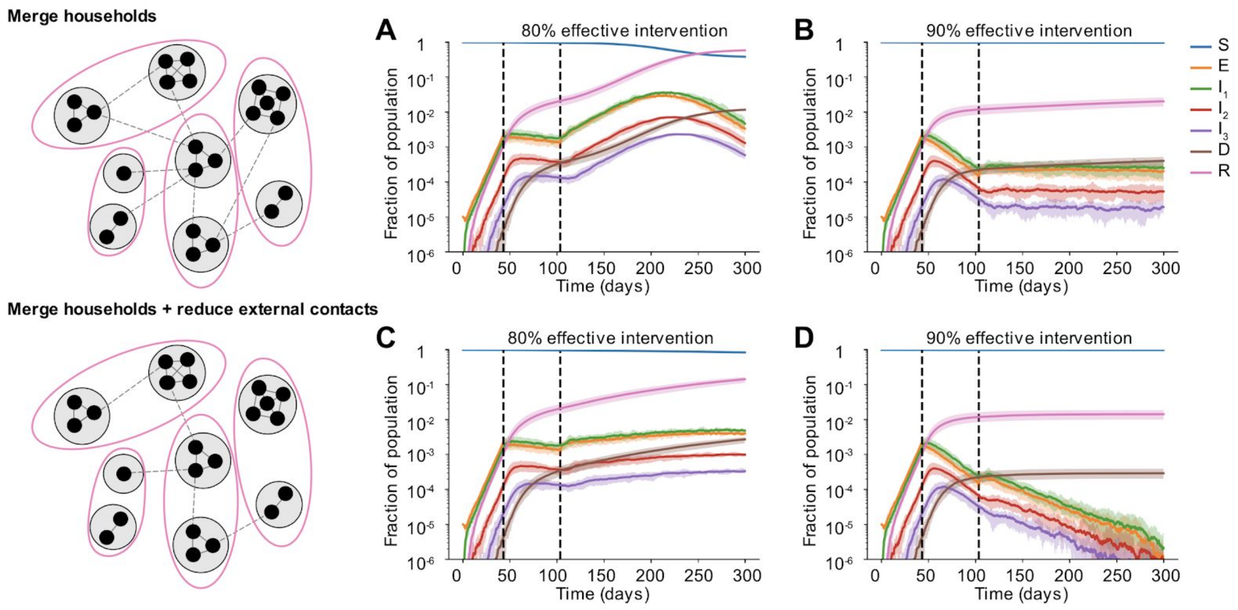

Figure S5: Effect of partially relaxing intervention by forming household bubbles, with neighborhood clustering. Some time after a social distancing intervention was implemented, each household merges with another random household irrespective of their "neighborhood". In each resulting two-household "bubble", all individuals are connected to all other individuals. A)-D) Simulated time courses of infection before and after social distancing interventions (with 80% vs 90% intervention efficacy) and after partial-relaxation by household merging. Top row: External contacts of individuals were unchanged after two households were merged, such that overall number of contacts increased. Bottom row: External contacts for individuals were reduced after two households were merged, such that overall number of contacts remained unchanged. In all cases, intervention was started 43 days after the onset of the epidemic (first black dotted line) and was relaxed after two months (60 days, second black dotted line).

medRxiv preprint doi: https://doi.org/10.1101/2020.06.04.20121673; this version posted January 15, 2021. The copyright holder for this preprint
(which was not certified by peer review) is the author/funder, who has granted medRxiv a license to display the preprint in perpetuity.
It is made available under a CC-BY 4.0 International license .

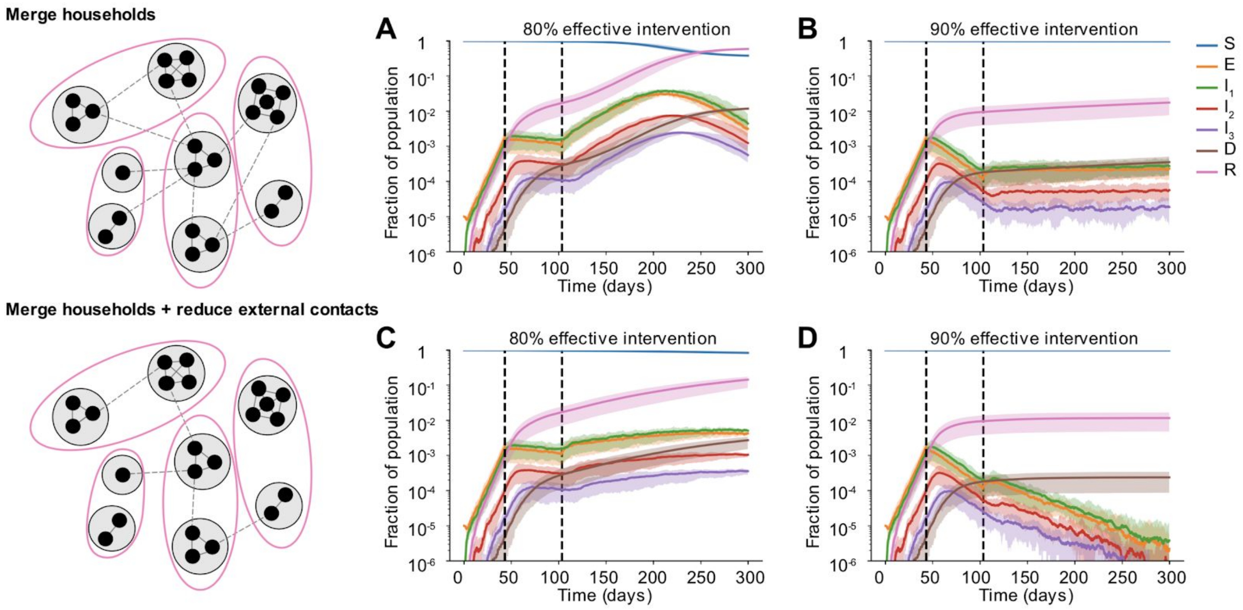

Figure S6: Effect of partially relaxing intervention by forming household bubbles, with neighborhood clustering. Some time after a social distancing intervention was implemented, each household merges with another household from their own "neighborhood". In each resulting two-household "bubble", all individuals are connected to all other individuals. A)-D) Simulated time courses of infection before and after social distancing interventions (with 80% vs 90% intervention efficacy) and after partial-relaxation by household merging. Top row: External contacts of individuals were unchanged after two households were merged, such that overall number of contacts increased. Bottom row: External contacts for individuals were reduced after two households were merged, such that overall number of contacts remained unchanged. In all cases, intervention was started 43 days after the onset of the epidemic (first black dotted line) and was relaxed after two months (60 days, second black dotted line).

medRxiv preprint doi: https://doi.org/10.1101/2020.06.04.20121673; this version posted January 15, 2021. The copyright holder for this preprint (which was not certified by peer review) is the author/funder, who has granted medRxiv a license to display the preprint in perpetuity.
It is made available under a CC-BY 4.0 International license .

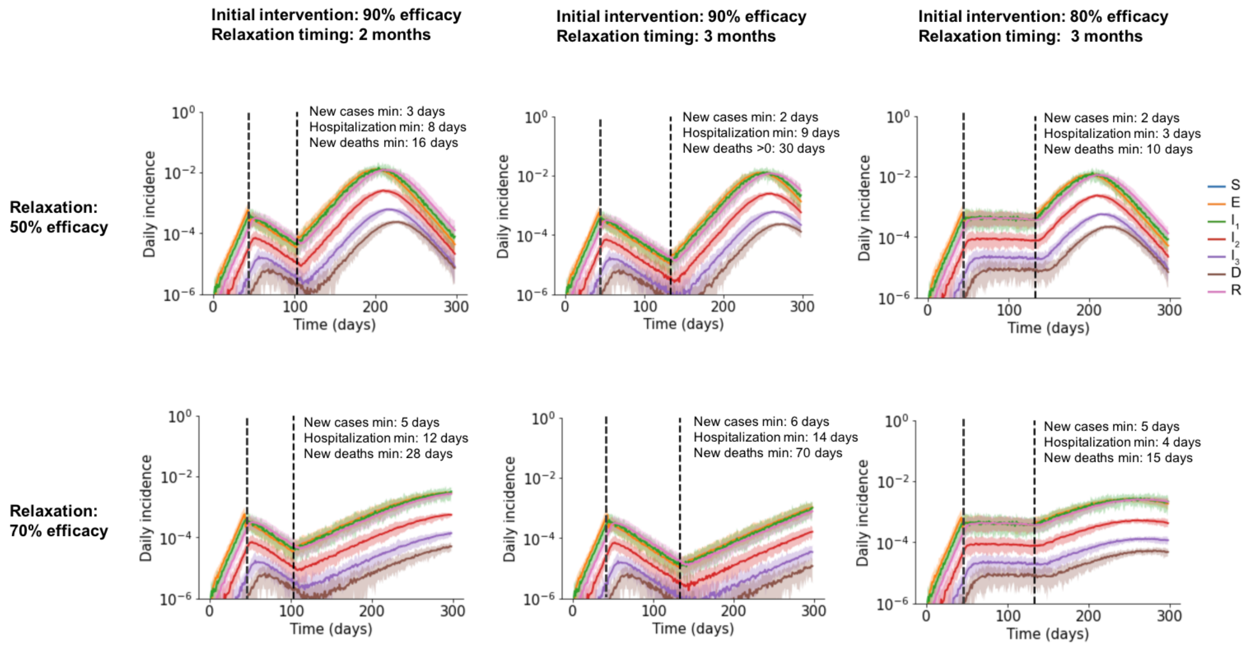

Figure S7. Effects of generalized relaxation of social distancing measures. Simulated time course of different clinical stages of infection under an initial intervention that reduces contacts (first black dashed line) and subsequent relaxation of that intervention (second black dashed line). Plots show daily incidence as a fraction of the total population. Solid line is mean and shaded areas are 5th and 95th percentile. Results are shown for different values of the efficacy of the initial intervention, efficacy during relaxation, and the timing of relaxation. The efficacy of the initial intervention and relaxation are defined as the % reduction in external contacts. In all plots, household and external contacts have equal weight. Plot annotations report the median time post-relaxation until different metrics of disease burden begin to increase - the daily incidence of new cases, current hospitalizations, or daily deaths. If deaths go to zero under an intervention, we instead report median time post-intervention to first death.

medRxiv preprint doi: https://doi.org/10.1101/2020.06.04.20121673; this version posted January 15, 2021. The copyright holder for this preprint
(which was not certified by peer review) is the author/funder, who has granted medRxiv a license to display the preprint in perpetuity.
It is made available under a CC-BY 4.0 International license .

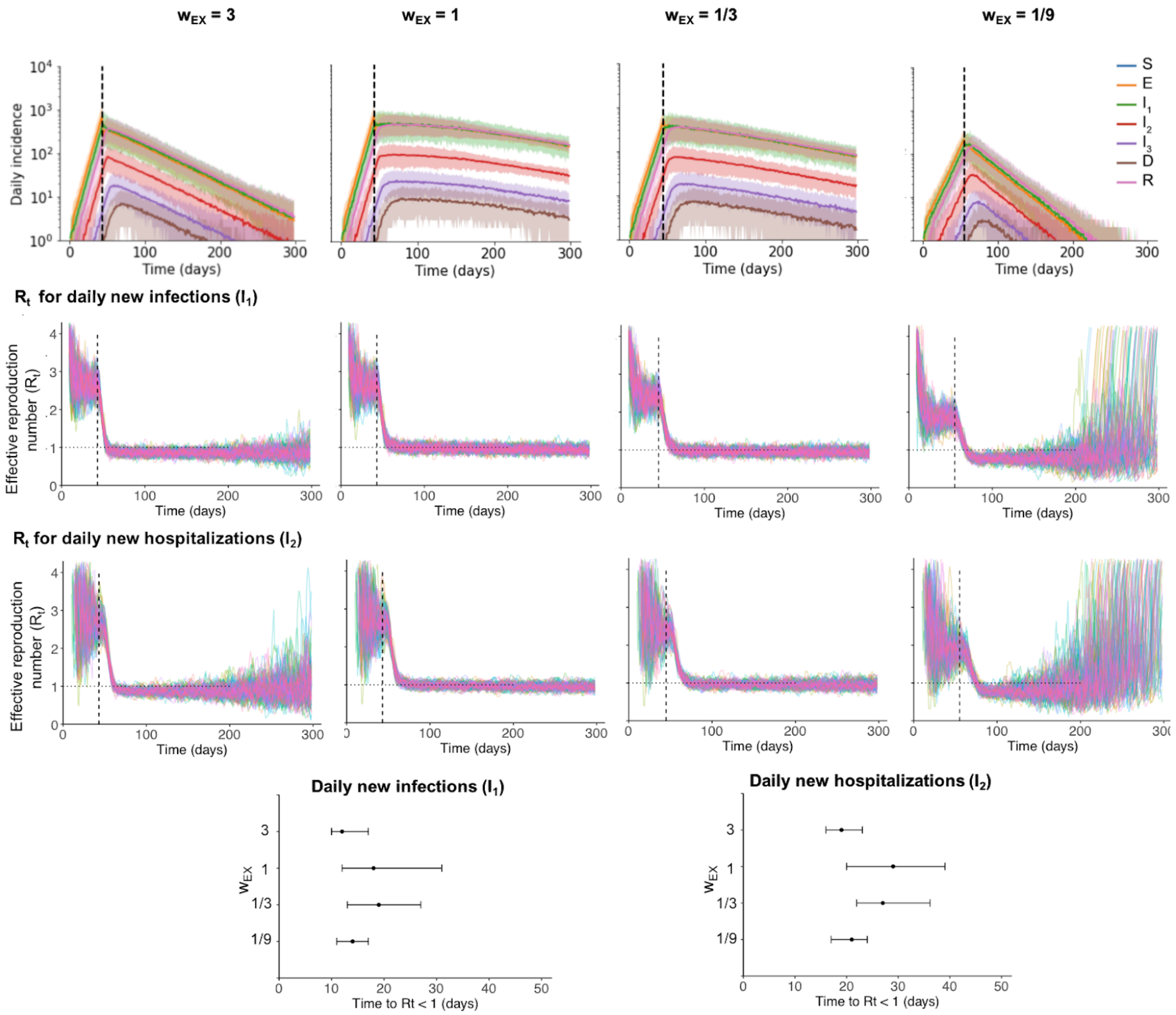

Figure S8. Time-varying effective reproduction number under social distancing. Top row: Simulated time course of different clinical stages of infection under an intervention (first black dashed line) that reduces external contacts by 80%, for different values of the relative weight of external contacts. Plots show daily incidence (for a total population of 1 million). Solid line is mean and shaded areas are 5th and 95th percentile of 100 simulations. Second row: Effective reproduction number (R_t) over time calculated using the daily incidence of new infections (I_1) for each simulation, calculated using the EpiEstim method with a 7-day window. The R_t value plotted for day t is calculated using timepoints before t only. Third row: Same but using the daily incidence of new hospitalizations (I_2). Bottom row: The first timepoint after the intervention that R_t <1. Dots show the median and bars represent 5th and 95th percentiles.
